# Predictors of serious adverse events and non‐response in cirrhotic patients with primary biliary cholangitis treated with obeticholic acid

**DOI:** 10.1111/liv.15386

**Published:** 2022-08-23

**Authors:** Antonio De Vincentis, Daphne D'Amato, Laura Cristoferi, Alessio Gerussi, Federica Malinverno, Ana Lleo, Francesca Colapietro, Fabio Marra, Andrea Galli, Cecilia Fiorini, Barbara Coco, Maurizia Brunetto, Grazia Anna Niro, Rosa Cotugno, Carlo Saitta, Raffaele Cozzolongo, Francesco Losito, Edoardo Giovanni Giannini, Sara Labanca, Marco Marzioni, Giulia Marconi, Anna Morgando, Rinaldo Pellicano, Ester Vanni, Nora Cazzagon, Annarosa Floreani, Luchino Chessa, Olivia Morelli, Luigi Muratori, Adriano Pellicelli, Maurizio Pompili, Francesca Ponziani, Annalisa Tortora, Floriano Rosina, Maurizio Russello, Mariarita Cannavò, Loredana Simone, Silvia Storato, Mauro Viganò, Ludovico Abenavoli, Maria D'Antò, Elisabetta De Gasperi, Marco Distefano, Gaetano Scifo, Teresa Zolfino, Vincenza Calvaruso, Giuseppe Cuccorese, Valeria Pace Palitti, Rodolfo Sacco, Gaetano Bertino, Evelise Frazzetto, Domenico Alvaro, Giacomo Mulinacci, Andrea Palermo, Miki Scaravaglio, Francesca Terracciani, Giovanni Galati, Vincenzo Ronca, Massimo Zuin, Ernesto Claar, Antonio Izzi, Antonio Picardi, Pietro Invernizzi, Umberto Vespasiani‐Gentilucci, Marco Carbone, on behalf of the Club Epatologi Ospedalieri (CLEO)/Associazione Italiana Gastroenterologi ed Endoscopisti Digestivi Ospedalieri (AIGO) PBC Study Group and, the Italian PBC Registry, Valentina Feletti, Alessandro Mussetto, Rosanna Venere, Giulia Bernaccioni, Marie Graciella Pigozzi, Stefano Fagiuoli, Natalia Terreni, Pietro Pozzoni, Leonardo Baiocchi, Giuseppe Grassi, Maria Vinci, Valentina Bellia, Roberto Boldizzoni, Silvia Casella, Barbara Omazzi, Guido Poggi

**Affiliations:** ^1^ Internal Medicine and Hepatology University Campus Bio‐Medico of Rome Rome Italy; ^2^ Gastroenterology Unit, Città della salute e della scienza Turin Italy; ^3^ Division of Gastroenterology, Centre for Autoimmune Liver Diseases, Department of Medicine and Surgery University of Milano‐Bicocca, European Reference Network on Hepatological Diseases (ERN RARE‐LIVER), San Gerardo Hospital Monza Italy; ^4^ Internal Medicine and Hepatology, Humanitas Clinical and Research Center IRCCS Humanitas University Milan Italy; ^5^ Internal Medicine and Hepatology Unit, Department of Experimental and Clinical Medicine University of Firenze Firenze Italy; ^6^ Department of Experimental and Clinical Biochemical Sciences University of Florence Florence Italy; ^7^ Hepatology Unit, University Hospital of Pisa Pisa Italy; ^8^ Gastroenterology Unit, Fondazione Casa Sollievo Della Sofferenza IRCCS San Giovanni Rotondo Italy; ^9^ Division of Medicine and Hepatology University Hospital of Messina “Policlinico G. Martino” Messina Italy; ^10^ Gastroenterology Unit National Institute of Gastroenterology “S de Bellis” Research Hospital Castellana Grotte Italy; ^11^ Gastroenterology Unit, Department of Internal Medicine University of Genova, IRCCS Ospedale Policlinico San Martino Genova Italy; ^12^ Clinic of Gastroenterology and Hepatology Università Politecnica delle Marche Ancona Italy; ^13^ Gastroenterology Unit, Department of Surgery, Oncology and Gastroenterology Padua University Hospital Padua Italy; ^14^ Liver Unit, University Hospital of Cagliari Cagliari Italy; ^15^ Clinic of Gastroenterology and Hepatology, Department of Medicine Università degli Studi di Perugia Perugia Italy; ^16^ DIMEC Università di Bologna, Policlinico di Sant'Orsola Bologna Italy; ^17^ Liver Unit, San Camillo Hospital Rome Italy; ^18^ Internal Medicine and Hepatology Unit, Policlinico Gemelli Sapienza University Rome Italy; ^19^ Medical Team Torino Torino Italy; ^20^ Liver Unit, Arnas Garibaldi Catania Italy; ^21^ Department of Gastroenterology University Hospital Sant'Anna Ferrara Italy; ^22^ IRCCS Sacro Cuore Institute Don Calabria, Gastroenterology Negrar Italy; ^23^ Hepatology Unit, San Giuseppe Hospital Milan Italy; ^24^ Department of Health Sciences University “Magna Graecia” of Catanzaro Italy; ^25^ Hepatology Unit, Santa Maria delle Grazie Hospital Pozzuoli Italy; ^26^ Foundation IRCCS Ca’ Granda Ospedale Maggiore Policlinico – Division of Gastroenterology and Hepatology – CRC “A.M. and A. Migliavacca” Center for Liver Disease Milan Italy; ^27^ Department of Infectious Diseases Umberto I Hospital Syracuse Italy; ^28^ Department of Gastroenterology Brotzu Hospital Cagliari Italy; ^29^ Gastroenterology and Hepatology Unit University of Palermo Palermo Italy; ^30^ Internal Medicine Ospedale “R. Dimiccoli” Barletta Italy; ^31^ Hepatology Unit, Santo Spirito Hospital Pescara Italy; ^32^ Gastroenterology Unit, Ospedali Riuniti Foggia Italy; ^33^ Gastroenterology and Hepatology Unit University Hospital Policlinico Vittorio Emanuele Catania Italy; ^34^ Department of Translational and Precision Medicine University La Sapienza Rome Italy; ^35^ Liver and Gastroenterology Unit, Department of Health Sciences Universita’ degli Studi di Milano Milan Italy; ^36^ ASST Santi Paolo e Carlo University Hospital San Paolo Milan Italy; ^37^ Department of Hepatology Betania Hospital Napoli Italy; ^38^ Department of Infectious Diseases D. Cotugno Hospital Napoli Italy

**Keywords:** decision curve analysis, efficacy, liver decompensation, safety, total bilirubin

## Abstract

**Background & Aims:**

Obeticholic acid (OCA) has recently been restricted in patients with primary biliary cholangitis (PBC) with “advanced cirrhosis” because of its narrow therapeutic index. We aimed to better define the predicting factors of hepatic serious adverse events (SAEs) and non‐response in cirrhotic patients undergoing OCA therapy.

**Methods:**

Safety and efficacy of treatment were evaluated in a cohort of consecutive PBC cirrhotic patients started with OCA. OCA response was evaluated according to the Poise criteria. Risk factors for hepatic SAEs and non‐response were reported as risk ratios (RR) with 95% confidence intervals (CIs).

**Results:**

One hundred PBC cirrhotics were included, 97 Child‐Pugh class A and 3 class B. Thirty‐one had oesophageal varices and 5 had a history of ascites. Thirty‐three per cent and 32% of patients achieved a biochemical response at 6 and 12 months respectively. Male sex (adjusted‐RR 1.75, 95%CI 1.42–2.12), INR (1.37, 1.00–1.87), Child‐Pugh score (1.79, 1.28–2.50), MELD (1.17, 1.04–1.30) and bilirubin (1.83, 1.11–3.01) were independently associated with non‐response to OCA. Twenty‐two patients discontinued OCA within 12 months: 10 for pruritus, 9 for hepatic SAEs (5 for jaundice and/or ascitic decompensation; 4 for upper digestive bleeding). INR (adjusted‐RR 1.91, 95%CI 1.10–3.36), lower albumin levels (0.18, 0.06–0.51), Child‐Pugh score (2.43, 1.50–4.04), history of ascites (3.5, 1.85–6.5) and bilirubin (1.30, 1.05–1.56), were associated with hepatic SAEs. A total bilirubin≥1.4 mg/dl at baseline was the most accurate biochemical predictor of hepatic SAEs under OCA.

**Conclusions:**

An accurate baseline assessment is crucial to select cirrhotic patients who can benefit from OCA. Although OCA is effective in one third of cirrhotics, bilirubin level ≥1.4 mg/dl should discourage from its use.

**Key points**

Treatment guidelines recommend that patients with primary biliary cholangitis (PBC) who have an inadequate response, or are intolerant, to ursodeoxycholic acid consider obeticholic acid (OCA) as second‐line therapy. Notably, patients with cirrhosis were poorly represented in the pre‐marketing clinical trials with OCA. Recently, based on a small series of scattered reports concerning hepatic decompensation during OCA treatment in PBC patients with cirrhosis, the Food and Drug Administration (FDA) has restricted the use of the drug in PBC patients having “advanced cirrhosis”. Here, by analysing a real‐world cohort of 100 cirrhotic patients with PBC followed for 12 months after the beginning of OCA treatment, we were able to extract rates of biochemical response, of OCA discontinuation, of pruritus and of hepatic severe adverse events (SAEs). We also identified some clinical predictors of hepatic SAEs, among which a total bilirubin≥1.4 mg/dl emerged as the most accurate. Our results suggest that OCA treatment is still effective in cirrhotic patients but an accurate baseline assessment is crucial in order to maximize its benefit/risk ratio.

## 1 INTRODUCTION

Primary biliary cholangitis (PBC) is an autoimmune disease of the small‐ and medium‐size bile ducts causing chronic cholestasis, which, if untreated or undertreated, can slowly progress to liver fibrosis and cirrhosis.^1^ Ursodeoxycholic acid (UDCA) is the first‐line treatment and is effective in ~60% of patients, depending on the definition of treatment response applied.^2^, ^3^, ^4^ Obeticholic acid (OCA) is the registered second‐line treatment which is offered to patients who do not achieve a satisfactory response, or are intolerant, to UDCA.^1^ The addition of OCA can rescue to response ~40% of UDCA non‐responders.^5^, ^6^ Several real‐world experiences have shown that OCA is however less effective in cirrhotic patients, in whom it is associated with a higher drop‐out rate from treatment because of higher occurrence of adverse events.^7^, ^8^, ^9^

Recently, the Food and Drug Administration (FDA) has restricted the use of OCA in PBC patients having “advanced cirrhosis”^10^ based on the report of 25 PBC cirrhotic patients, with either compensated and decompensated cirrhosis before starting OCA, who developed serious liver injury leading to liver decompensation or liver failure under OCA treatment. Notably, this restriction has been quickly incorporated into the PBC guidelines of the American Association for the Study of Liver Diseases (AASLD).^11^ The FDA, therefore, recommends that, before starting OCA, health care professionals should determine whether a patient with PBC has “advanced cirrhosis”, generically defined as cirrhosis with current or prior evidence of hepatic decompensation or portal hypertension.^10^

However, an accurate definition of predicting factors for decompensation under OCA for PBC cirrhotic patients is lacking. This may expose some to a possibly harmful treatment and, on the other hand, deprive some others of effective therapy in a stage of disease where it is highly needed.

In this study using data from the *Italian PBC Registry*, we aimed to verify the efficacy and safety profile of OCA therapy in a large cohort of PBC cirrhotic patients, and to identify biochemical predictors of hepatic severe adverse events (SAEs) and non‐response enabling a more accurate selection for OCA therapy in this at‐risk category of PBC patients.

## 2 METHODS

### 2.1 Study design and participants

This is a multicenter, observational study carried out within the *Italian PBC Registry*, an ongoing, non‐interventional, multicenter, retrospective and prospective, observational cohort study that monitors PBC patients enrolled in all Italian centres following PBC patients (33 centres). All adult patients who had received a diagnosis of PBC at cirrhotic stage, who have started OCA taking at least 1 dose of the drug, and who had an overall follow‐up of at least 12 months (therefore, having started OCA not later than May 2020), were included in the study. Notably, all patients withdrawing OCA for different reasons remained under follow‐up in the cohort study.

Indications to OCA treatment, which in Italy essentially coincides with the criteria by which second‐line therapy with OCA is reimbursed by the National Health Service, were an alkaline phosphatase (ALP) ≥1.5 per upper limit of normal (ULN) and/or 1 mg/dl≤bilirubin≤2 mg/dl after at least 12 months of treatment with UDCA, or the intolerance to UDCA. According to the package insert in Italy, in patients with compensated cirrhosis, OCA therapy should be initiated at 5 mg/day dose and re‐evaluated after 6 months for possible up‐titration to 10 mg/day in case of suboptimal response, where suboptimal response is not further defined but generally assumed to be an ALP level still ≥1.5/ULN. Conversely, in patients with decompensated liver disease (Child‐Pugh B and C cirrhosis), OCA is recommended to be started at 5 mg/week dose, and, if tolerated and judged necessary according to suboptimal response after 3 months, gradually up‐titrated until a maximum dose of 10 mg twice weekly.

Exclusion criteria were having been previously enrolled in a sponsored trial with OCA and being on off‐label fibrate therapy not on stable regimen for at least 6 months at the time of OCA start. The study was conducted in accordance with the Declaration of Helsinki guidelines and the principles of good clinical practice. All participants to the Italian PBC Registry provided written informed consent. The study was approved by the University of Milan‐Bicocca research ethics committee (Study name: PBC322), coordinator of the Italian National Registry and by the Research and Development Department of each collaborating hospital.

### 2.2 Data capture

Data were captured using baseline and follow‐up case record forms (CRFs), completed by physicians in each collaborating centre. Demographic, clinical and biochemical data were collected at baseline (immediately before starting OCA therapy), and at 6 and 12 months of treatment during follow‐up visits. The model for end‐stage liver disease (MELD) and the Child‐Pugh score were computed. Management of OCA therapy was tailored on each patient and clinical decisions were taken independently by each physician based only on drug package insert. Data on OCA dose adjustment and OCA discontinuation were collected. Pruritus was systematically assessed at baseline and at every follow‐up visit. Other adverse events were not systematically assessed but registered when they led to permanent drug discontinuation. Completed CRFs underwent quality control for completeness and accuracy at the University of Milan‐ Bicocca, Milan and University Campus Bio‐Medico, Rome. Missing, inaccurate or implausible data were systematically queried with the treating physicians. Data that passed quality control were uploaded into a bespoke database, collecting clinical and biochemical data at each follow‐up time. The database is an electronic data capture (EDC) system with e‐CRF developed for the purpose of this study and the other projects on the Italian PBC Registry. The EDC system runs on a server maintained by a dedicated Clinical Research Organization (CRO). The EDC system allows research staff in collaborating centres to log into it from any National Health Service (NHS) computer to view information about participants recruited from their own centres and to complete e‐CRFs and upload the results of medical investigations directly into the database.

### 2.3 Study definitions

#### 2.3.1 Diagnosis

PBC was diagnosed according to the European Association for the Study of the Liver (EASL) criteria^1^: co‐existence of elevated cholestatic serum biomarkers (ALP, γ‐glutamyl‐transferase (GGT) and bilirubin) and anti‐mitochondrial antibodies (AMA) or specific anti‐nuclear antibodies (ANA sp100 and gp210). Liver biopsy was performed to confirm the diagnosis for AMA‐negative PBC and for PBC – autoimmune hepatitis (AIH) overlap syndrome. All PBC—AIH patients included in the study were on a stable immunosuppressive treatment for at least 6 months.

Liver cirrhosis was defined by either: (1) liver histology; and/or (2) liver stiffness by vibration‐controlled transient elastography≥16.9 kPa; and/or (3) the presence of some ultrasonographic findings that, in the context of chronic liver disease, have been proven to be highly specific for liver cirrhotic evolution, that is, liver surface nodularity with−/out caudate lobe hypertrophy among morphologic signs, and/or increased portal diameter with portal flow velocity reduction and/or presence of porto‐systemic collaterals among signs of portal hypertension.^12^, ^13^, ^14^

#### 2.3.2 Study cohort

We defined the overall cohort (OC) as all patients who had received at least one dose of OCA and had at least 12 months of follow‐up; and the treatment completer cohort (TCC) as all patients completing the treatment period of 6 or 12 months for the analysis at 6 or 12 months respectively.

#### 2.3.3 Response

The biochemical response to OCA therapy was evaluated at 6 and 12 months and in both the OC and TCC, according to the following two criteria: (1) ALP <1.67/ULN with a reduction of ≥15% from baseline and a normal total bilirubin level, as applied in the registrative trial of OCA (*Poise criteria*); (2) ALP, alanine aminotransferase (ALT) and bilirubin within the normal range (*normal range criteria*), since normalization of liver biochemistry has been recently proposed as a new therapeutic target in PBC.^15^

#### 2.3.4 Study endpoints

Occurrence of biochemical response and/or hepatic SAEs; predictors of non‐response and of hepatic SAEs.

### 2.4 Statistical analysis

Continuous variables were expressed as median with interquartile range (IQR), whereas categorical ones with absolute frequencies and percentages. The χ^2^ test and the Wilcoxon test were applied for group comparisons, as appropriate. To account for inter‐laboratory variability, ALP, GGT, ALT and aspartate aminotransferase (AST), and total bilirubin were expressed as ratios of their respective ULN. The analysis of risk factors for no response after 12 months of OCA therapy and for the occurrence of liver decompensation was carried out by reporting risk ratios (RR) with 95% CIs, and performed by means of Poisson regression models with robust error variance, as described by Zou et al.^16^ Multivariable analyses included all significantly and nearly significantly associated variables, that is, those with a *p* value <.10 at univariate analysis.

Then, the discriminative capacities of the main risk factors for hepatic SAEs were derived by computing the area under the receiver operating characteristic (AUROC) curve for continuous variables, and by reporting general accuracy, sensitivity, specificity, positive (PPV) and negative predictive values (NPV) for categorical variables. The best performing cut‐offs of continuous variables were extracted for the ROC curves using the Youden method, and were reported accordingly.

Finally, decision curve analysis (DCA) was performed on the OC to compute the net benefit of decisions to treat cirrhotic PBC subjects with OCA based on different clinical and biochemical parameters (e.g., total bilirubin, albumin, MELD, Child‐Pugh score, etc.), measured at baseline.

The net benefit was estimated as the rate that incorporating the decision guide of interest (such as total bilirubin or albumin, etc.) would lead to additional beneficial decision to treat cirrhotic PBC subjects without causing any additional harmful decision to overtreat the disease. The net benefit is computed and plotted across a range of threshold probabilities, defined as the minimum probability of treatment success that can be accepted. Moreover, the net benefit of each strategy is compared with that achieved by two default strategies, that is, those of *treat none* and *treat all* patients. By definition, the threshold probability at the intersection of the *treat all* and the *treat none* lines represent the baseline probability of treatment success without implementing any other parameter in the decision‐making strategy. The net reduction of OCA therapies based on the different parameter was also reported as the mean number of saved therapies per 100 prescription with standard deviation (SD). DCA was performed using publicly available code^17^ and the R statistics package. More details on DCA are reported as Supplementary Materials. All analyses were undertaken using R version 4.0.2 (R Foundation for Statistical Computing, Vienna, Austria; https://www.R‐project.org/).

## 3 RESULTS

### 3.1 Characteristics of the study cohort

Out of 106 cirrhotic PBC patients from the *Italian PBC Registry* who had been prescribed OCA between September 1st 2017 and May 1st 2021, 6 were excluded because they had not completed 12 months of therapy (starting OCA after May 1st 2020). One hundred subjects (median age 62 years, 95% women) were included in the analyses. The general characteristics of the study cohort are reported in Table 1. Notably, only 3 subjects had a Child‐Pugh class B and the median MELD was 6.9 (interquartile range, IQR, 6.4–8.5). Thirty‐one patients had oesophageal varices and 5 had a history of previous ascites, 4 in complete remission with diuretics and one with mild residual ascites at the time of OCA start. None of the patients had history of upper digestive bleeding or hepatic encephalopathy. In all cases, OCA therapy was indicated for inadequate response after at least 12 months of UDCA, and 98% of subjects were non‐responders according to Paris II criteria. The majority of patients (65%) were prescribed and took OCA 5 mg daily for all the therapy course, whereas 20% started OCA 5 mg daily and then were up‐titrated to 10 mg daily. Overall, 15 patients were prescribed with OCA less than 5 mg daily. Ten patients were under triple therapy with UDCA, OCA and fibrates.

**TABLE 1:** General characteristics of the study cohort

Characteristic	*N* = 100
Socio‐demographics and comorbidities	
Sex, female	95 (95%)
Age at OCA start, years	62 (54, 67)
Age at PBC diagnosis, years	52 (43, 56)
Duration of disease before OCA start, years	9 (5, 15)
Body Mass Index, Kg/m^2^	24.6 (22.0, 26.9)
Diabetes mellitus	12 (12%)
Liver disease characterization	
AMA positivity	83 (83%)
ANA positivity	52 (52%)
PBC‐AIH overlap	14 (14%)
Diagnosis of cirrhosis	
Clinical^a^	69 (69%)
Histological	24 (24%)
Elastographic^b^	7 (7%)
Child‐Pugh class	
A^c^	97 (97%)
B	3 (3%)
C	0 (0%)
MELD	6.9 (6.4, 8.5)
Ascites	
Absent	95 (95%)
Controlled with diuretics	4 (4%)
Present	1 (1%)
Hepatic encephalopathy	0 (0%)
Oesophageal varices, presence	31 (31%)
Gastroscopy not performed	16 (16%)
OCA therapy	
Indication to OCA start	
UDCA intolerance	0 (0%)
Inadequate response to UDCA	100 (100%)
acc. to Paris I criteria	60 (60%)
acc. to Paris II criteria	98 (98%)
acc. to Toronto criteria	79 (79%)
OCA regimen	
5 mg daily	65 (65%)
5 mg daily uptitrated to 10 mg	20 (20%)
5 mg every other day uptitrated to 5 mg daily	4 (4.0%)
5 mg weekly	4 (4.0%)
5 mg 3 times a week uptitrated to 5 mg daily	3 (3.0%)
5 mg every other day	2 (2.0%)
5 mg twice a week	2 (2.0%)
Concomitant/Previous therapies	
UDCA dose, mg/kg	15.00 (15.00, 17.04)
Fibrate therapy	
NO	81 (81%)
Before and stopped before OCA start	8 (8.0%)
Before and continued during OCA therapy	7 (7.0%)
After OCA start	3 (3.0%)
After OCA discontinuation	1 (1.0%)
Biochemical	
ALP/ULN at baseline	2.10 (1.72, 2.89)
ALT/ULN at baseline	1.07 (0.78, 1.76)
AST/ULN at baseline	1.23 (0.90, 1.83)
GGT/ULN at baseline	4.5 (2.8, 7.0)
Total Bilirubin/ULN at baseline	0.90 (0.70, 1.21)
Platelets (x10^9^/L)	152 (120, 206)
Albumin, g/dl	4.00 (3.60, 4.24)
INR	1.00 (0.97, 1.10)
Creatinine, mg/dl	0.70 (0.60, 0.80)

*Note*: Data reported as median with interquartile range or as numbers with percentages.

Paris I criteria: ALP <3x ULN, ALT <2x ULN and bilirubin <1 mg/dl. Paris II criteria: ALP <1.5x ULN, ALT <1.5x ULN and bilirubin <1 mg/dl. Toronto criteria: ALP <1.67x ULN.

Abbreviations: Acc, according; AIH, autoimmune hepatitis; ALP, alkaline phosphatase; ALT, alanine transferase; AMA, antimitochondrial antibodies; ANA, antinuclear antibodies; AST, aspartate transferase; GGT, gamma‐glutamyl transferase; INR, international normalized ratio; MELD, model for end‐stage liver disease; PBC, primary biliary cholangitis; UDCA, ursodeoxycholic acid; ULN, upper limit of normal.

^a^ By ultrasonography, all these 69 subjects had morphologic signs specific for liver cirrhosis, and 41 had also ultrasonographic signs specific for portal hypertension (as specified in Methods section).

^b^ Fibroscan ≥16.9 KPa.

^c^ Out of 97 subjects with Child‐Pugh class A, 80 and 17 had a Child‐Pugh score of 5 and 6 respectively.

### 3.2 Response rate to treatment at 6 and 12 months

**FIGURE 1:**
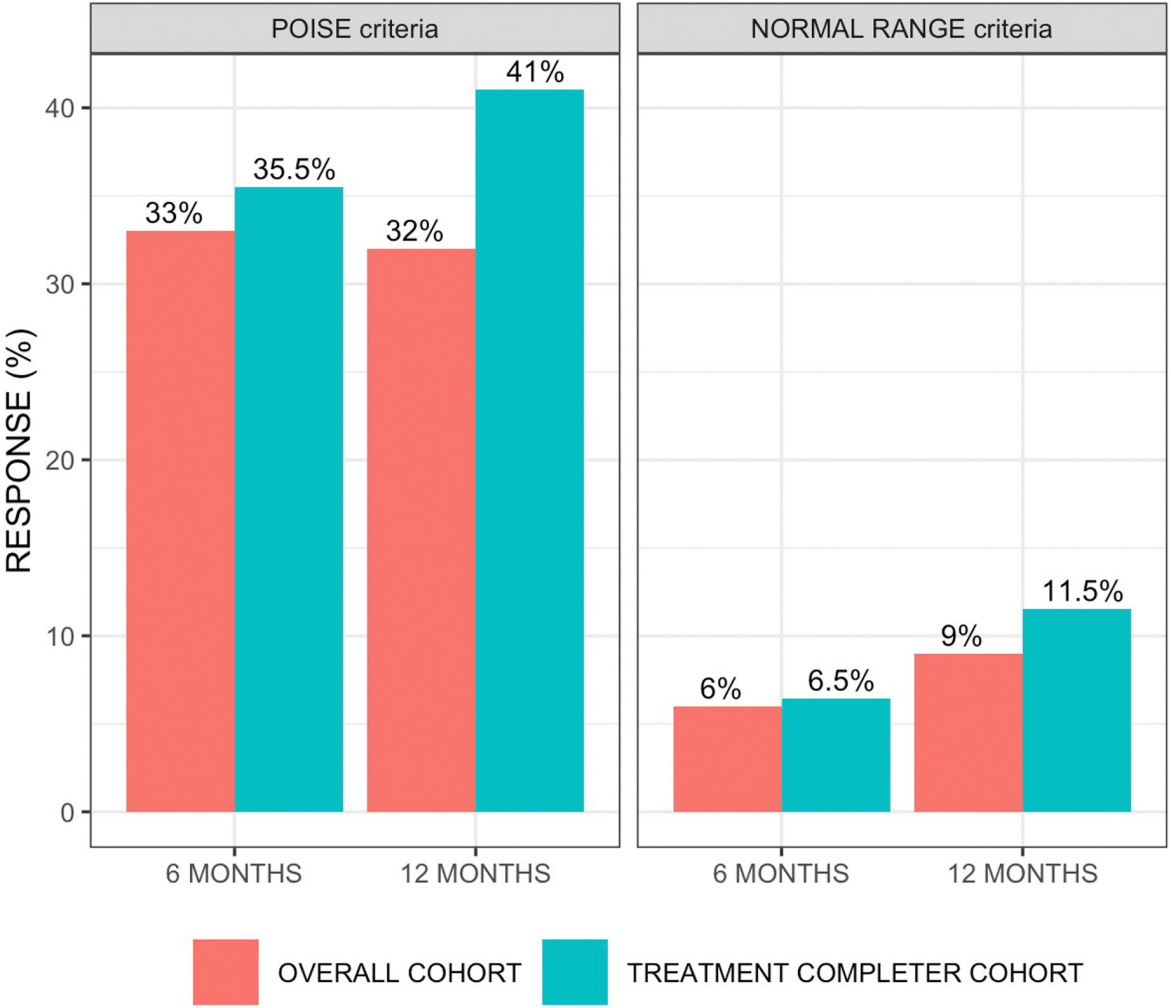
Rates of response to Obeticholic acid therapy according to the POISE (*left panel*) and the normal range criteria (*right panel*) in the overall cohort and the treatment completer cohort.

According to the *Poise criteria*, 33% and 32% of patients achieved a response at 6 and 12 months, respectively, in the OC population; and 35.5% and 41% at 6 and 12 months, respectively, in the TCC population (Figure 1). According to the *normal range criteria*, 6% and 9% of patients achieved a response at 6 and 12 months, respectively, in the OC population; and 6.5% and 11.5% at 6 and 12 months, respectively, in the TCC population (Figure 1). Considering only the 79 patients with a baseline ALP/ULN≥1.67, 27.8% and 5.1% in the OC, while 36.1% and 6.6% in the TCC achieved a response at 12 months, according to *Poise* and *normal range criteria*, respectively (*p* > .05 for all comparisons with OCA response rate in the complete cohort; Figure S1). Progressive reduction of ALP and ALT were observed at 6 and 12 months, while substantial stability of total bilirubin levels was observed in the OC (Figure S2).

Male sex, history of ascites, albumin levels, Child‐Pugh score, MELD score, ALP/ULN, GGT/ULN, ALT/ULN, AST/ULN and total bilirubin, were significantly associated with a reduced probability of biochemical response to OCA therapy according to *Poise criteria* (Table 2). After correction, male sex (adjusted [a‐]RR for female sex 0.63, 95% CI 0.42–0.93), INR (aRR 1.37, 95%CI 1.00–1.87), Child‐Pugh score (aRR 1.79, 95%CI 1.28–2.50), MELD (aRR 1.17, 95%CI 1.04–1.30) and total bilirubin (aRR 1.83 95%CI 1.11–3.01) were independently associated with non‐response to OCA (Table 2).

**TABLE 2:** Factors associated with lack of response to Obeticholic acid at 12 months

Variable	Univariate			Multivariate		
	RR	95%CI	*p*	aRR	95%CI	*p*
Age at OCA start, years	1.00	0.98–1.02	.905			
Age at PBC diagnosis, years	1.00	0.99–1.02	.647			
Duration of PBC, years	0.99	0.97–1.02	.634			
Female sex	0.57	0.47–0.70	<.001	0.63	0.42–0.93	.020
Diabetes Mellitus	0.93	0.50–1.73	.831			
BMI, Kg/m^2^	0.95	0.90–1.01	.081	0.95	0.9–1.00	.070
ANA positivity	1.35	0.92–1.98	.125			
AMA positivity	0.75	0.51–1.10	.144			
PBC‐AIH overlap	0.99	0.59–1.66	.961			
Concomitant Fibrate therapy	1.38	0.92–2.07	.123			
Oesophageal varices	1.38	0.93–2.06	.110			
History of ascites^a^	1.71	1.42–2.07	<.001	0.91	0.48–1.73	.780
Platelets <150 000/mm^3^	1.10	0.75–1.62	.635			
INR^b^	1.18	0.98–1.42	.081	1.37	1.00–1.87	.048
Albumin, g/dl	1.01	1.01–1.02	<.001	1.01	1.00–1.02	.100
Creatinine, mg/dl	1.12	0.75–1.68	.578			
Child‐Pugh score	1.57	1.12–2.20	.008	1.79^d^	1.28–2.50	<.001
MELD	1.12	1.04–1.21	.003	1.17^d^	1.04–1.30	.004
OCA dose^c^	1.06	0.78–1.44	.720			
ALP/ULN at baseline	1.25	1.13–1.38	<.001	1.07	0.93–1.24	.340
ALT/ULN at baseline	1.23	1.04–1.45	.015	1.32	0.89–1.96	.170
AST/ULN at baseline	1.42	1.11–1.83	.006	0.77	0.42–1.40	.390
GGT/ULN at baseline	1.01	1.00–1.02	.006	1.02	1.00–1.04	.080
Total bilirubin at baseline	2.08	1.49–2.90	<.001	1.83	1.11–3.01	.020

*Note*: Lack of response to Obeticholic acid were evaluated according to Poise criteria in the treatment completer cohort (TTC). Risk ratios with 95% confidence intervals were from Poisson regression models with robust error variance. All variables associated at univariate analysis with a *p* < .10 entered the multivariate model.

Abbreviations: AIH, autoimmune hepatitis; ALP, alkaline phosphatase; ALT, alanine transferase; AMA, antimitochondrial antibodies; ANA, antinuclear antibodies; aRR, adjusted risk ratio; AST, aspartate transferase; BMI, body mass index; GGT, gamma‐glutamyl transferase; INR, international normalized ratio; MELD, model for end‐stage liver disease; OCA, obeticholic acid; PBC, primary biliary cholangitis; RR, risk ratio; UDCA, ursodeoxycholic acid; ULN, upper limit of normal.

^a^ currently controlled by diuretic therapy.

^b^ Risk estimates reported for 1 standard deviation increase to provide a more clinically useful result.

^c^ OCA dose categorized as <5 mg daily, 5 mg daily and >5 mg daily.

^d^ Since included in their computation, Child‐Pugh score and MELD were included in multivariate models after exclusion of ascites, albumin, INR and total bilirubin (for Child‐Pugh score) and INR and total bilirubin for MELD.

### 3.3 OCA treatment discontinuation

Twenty‐two patients interrupted OCA treatment before 12 months (Table 3), 32% and 68% of them before 6 and between 6 and 12 months respectively. Pruritus was the leading cause in 10 patients (45%). Nine patients discontinued OCA for hepatic SAEs; in particular, 5 developed jaundice and/or ascites, 3 patients had upper digestive bleeding, and 1 patient died after transjugular intrahepatic portosystemic shunt (TIPS) placement for refractory upper digestive bleeding.

**TABLE 3:** Occurrence of discontinuation of Obeticholic acid

Characteristic	*N* = 100
OCA discontinuation, *n*	22 (22%)
Time of OCA discontinuation	
Before 6 months	7 (32%)
Between 6 and 12 months	15 (68%)
Reason for OCA discontinuation	
Pruritus	10 (45.5%)
Hepatic severe adverse events^a^	9 (40.9%)
Anaemia	1 (4.5%)
Complication after hip fracture	1 (4.5%)
COVID‐19	1 (4.5%)

^a^ Includes patients experiencing on‐treatment death after TIPS placement for refractory bleeding from portal hypertensive gastropathy (1, 4.5%).

### 3.4 Hepatic SAEs during OCA treatment

In the OC, 9% of all PBC cirrhotic patients on OCA therapy experienced a hepatic SAE leading to OCA discontinuation before 12 months. These subjects had higher baseline median MELD (8.51 vs. 6.73, *p* .009), total bilirubin (1.4 vs. 0.9, *p* .015) and INR (1.12 vs. 1.00, *p* .05) and lower albumin levels (3.3 vs. 4.0, *p* = .003; Table S1). Notably, all the three patients with Child‐Pugh B class at baseline experienced hepatic SAEs.

At baseline, history of ascites, elevated INR, lower albumin levels, advanced Child‐Pugh score, elevated MELD and total bilirubin, and abnormal AST were associated with the risk of hepatic SAEs during OCA (Table 4). After appropriate adjustment, history of ascites (aRR 3.50, 95%CI 1.85–6.50), INR (1.91, 95%CI 1.10–3.36), lower albumin levels (aRR for albumin 0.18, 95%CI 0.06–0.51), Child‐Pugh score (aRR 2.43, 95%CI 1.50–4.04) and total bilirubin (aRR 1.30, 95%CI 1.05–1.56) were independently associated with hepatic SAEs (Table 4).

**TABLE 4:** Factors associated with hepatic severe adverse events during treatment with obeticholic acid

	Univariate			Multivariate		
Variable	RR	95%CI	*p*	aRR	95%CI	*p*
Age at OCA start, years	1.01	0.93–1.09	.855			
Age at PBC diagnosis, years	1.01	0.93–1.09	.854			
Duration of PBC, years	1.00	0.93–1.07	.947			
Female sex	0.42	0.06–2.74	.366			
Diabetes mellitus	0.92	0.13–6.70	.932			
BMI, kg/m^2^	0.92	0.79–1.08	.311			
ANA positivity	1.85	0.49–6.97	.366			
AMA positivity	0.72	0.16–3.16	.66			
PBC‐AIH overlap	0.77	0.10–5.68	.796			
Oesophageal varices	2.48	0.79–9.87	.123			
Concomitant fibrate therapy	1.12	0.16–8.10	.907			
History of ascites	4.54	2.67–7.72	<.001	3.50	1.85–6.50	<.001
Platelets <150 000 /mm^3^	0.59	0.16–2.23	.437			
INR^a^ ^,^ ^b^	2.11	1.25–3.56	.005	1.91	1.10–3.36	.024
Albumin, g/dl^b^	0.13	0.06–0.28	<.001	0.18	0.06–0.51	.001
Creatinine, mg/dl	1.52	0.37–6.18	.561			
Child‐Pugh score^c^	2.32	1.75–3.06	<.001	2.43	1.50–4.04	<.001
MELD^c^	1.32	1.15–1.50	<.001	1.23	1.09–1.39	<.001
OCA dose^d^	0.85	0.27–2.67	.775			
ALP/ULN at baseline	1.26	0.79–1.99	.331			
ALT/ULN at baseline^b^	1.66	0.98–2.83	.06	1.00	0.54–1.99	.918
AST/ULN at baseline^b^	1.82	1.40–2.35	<.001	0.91	0.58–1.43	.680
GGT/ULN at baseline	0.98	0.93–1.04	.574			
Total bilirubin at baseline^b^	1.53	1.34–1.74	<.001	1.30	1.05–1.56	.014

*Note*: Risk ratios with 95% confidence intervals were from Poisson regression models with robust error variance. All variables associated at univariate analysis with a *p* < .10 entered the multivariate model.

Abbreviations: AIH, autoimmune hepatitis; ALP, alkaline phosphatase; ALT, alanine transferase; AMA, antimitochondrial antibodies; ANA, antinuclear antibodies; aRR, adjusted risk ratio; AST, aspartate transferase; BMI, body mass index; GGT, gamma‐glutamyl transferase; INR, international normalized ratio; MELD, model for end‐stage liver disease; OCA, obeticholic acid; PBC, primary biliary cholangitis; RR, risk ratio; UDCA, ursodeoxycholic acid; ULN, upper limit of normal.

^a^ Risk estimates reported for 1 standard deviation increase to provide a more clinically useful result.

^b^ Alternatively included in multivariable models to avoid multiple collinearity.

^c^ Since included in their computation, Child‐Pugh score and MELD were included in multivariate models after exclusion of history of ascites, albumin, INR and total bilirubin (for Child‐Pugh score) and INR and total bilirubin for MELD.

^d^ OCA dose categorized as <5 mg daily, 5 mg daily and >5 mg daily.

**FIGURE 2:**
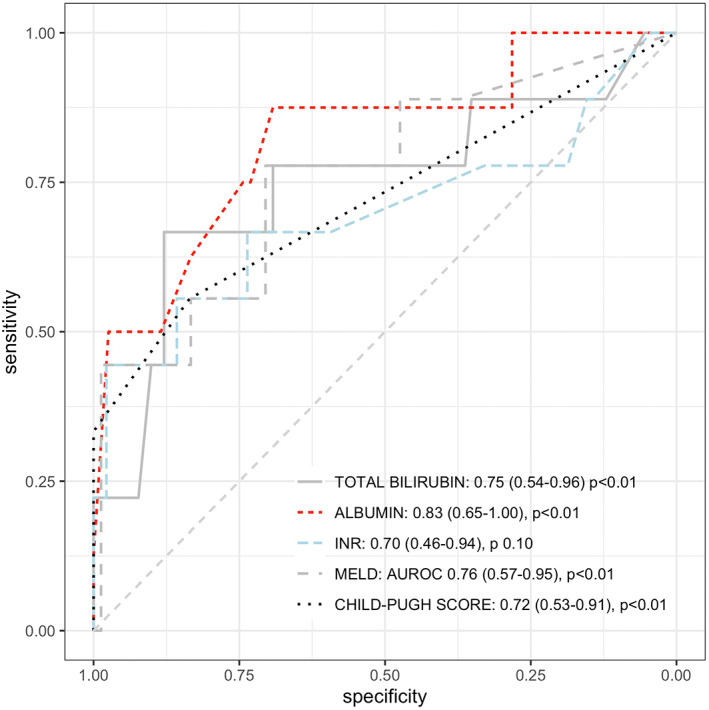
Discriminative capacities of factors associated with hepatic severe adverse events during Obeticholic acid treatment. Results are shown as area under the receiver operating characteristic, 95% confidence intervals and *p*‐values. Only predictors showing AUROC >0.70 are reported in the plot.

The discriminative capacities for hepatic SAEs of the main risk factors were reported in Figure 2. Overall, albumin levels, MELD, total bilirubin and Child‐Pugh score reported AUROCs of 0.83, 0.76, 0.75 and 0.72 respectively (*p* < .01 for all). Conversely, discrimination by INR was lower (AUROC 0.70, 95%CI 0.46–0.94, *p* .10). Total bilirubin levels of ≥1.4 mg/dL and history of ascites were the most accurate predictors of hepatic SAEs during OCA therapy (Table 5), mainly because of the high specificity and negative predictive value (accuracy, specificity and negative predictive value [NPV] of 0.86, 0.88 and 0.96 for total bilirubin and of 0.92, 0.98 and 0.94 for history of ascites), while sensitivity and positive predictive value (PPV) were lower (0.67 and 0.35 for total bilirubin, and 0.33 and 0.60 for history of ascites, respectively). Comparable NPV (~0.96–0.98) were observed also for albumin levels ≥3.7 g/L, Child‐Pugh score ≥6 or more and MELD ≥7.6, but with reduced overall accuracy (~0.71–0.81) and PPV (~0.23–0.25). Oesophageal varices and thrombocytopenia were per se not associated with the risk of hepatic SAEs. Indeed, among 42 patients with oesophageal varices and/or thrombocytopenia but bilirubin <1.4 mg/dl, only 2 (4.7%) underwent hepatic SAEs under OCA treatment. Conversely, among the 4 patients without oesophageal varices and thrombocytopenia but with bilirubin ≥1.4 mg/dl, 3 (75%) underwent hepatic SAEs while on OCA.

**TABLE 5:** Sensitivity, specificity, predictive values and general accuracy of main factors associated with hepatic SAEs during obeticholic acid treatment

	Cut‐off	Accuracy	Sensitivity	Specificity	PPV	NPV
Continuous variables						
Total bilirubin	≥1.4	0.86	0.67	0.88	0.35	0.96
Albumin	<3.7	0.71	0.88	0.69	0.23	0.98
Child‐Pugh score	≥6	0.81	0.56	0.84	0.25	0.95
MELD	≥7.6	0.71	0.78	0.71	0.23	0.96
Categorical variables						
History of ascites	Presence	0.92	0.33	0.98	0.60	0.94
Oesophageal varices	Presence	0.70	0.55	0.71	0.16	0.94

*Note*: Cut‐offs of continuous variables were selected applying the Youden method on the receiver operating characteristic (ROC) curves. Only continuous variable with area under the ROC (AUROC) >0.70 with a *p* < .05 (see Figure 2) were included.

Abbreviations: MELD, model for end‐stage liver disease; NPV, negative predictive value; PPV, positive predictive value.

### 3.5 Decision curve analysis

**FIGURE 3:**
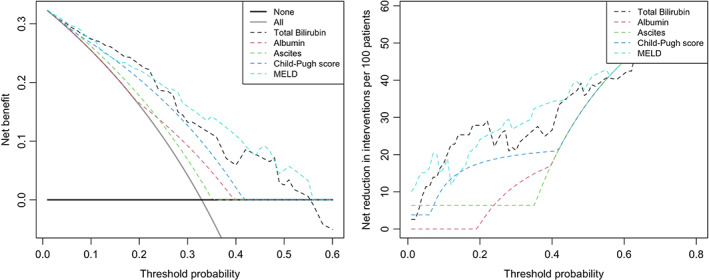
Decision curve analysis demonstrating the net benefit (*left panel*) and reduction of Obeticholic acid treatments (*right panel*), using different decision‐making strategies based on clinical and biochemical parameters. Threshold probability is the cut‐off probability of PBC subjects with liver cirrhosis at which an individual/physician considers the benefit of Obeticholic acid treatment equivalent to the harm of overtreatment, and thus reflects how the individual/physician weights the benefits and harms associated with this decision. The highest curve at any given threshold probability is the optimal decision‐making strategy to maximize net benefit.

Finally, a decision curve analysis (DCA) was performed to assess the clinical utility of using different biochemical and clinical variables to guide the decision of prescribing OCA in PBC patients with liver cirrhosis. As reported in Figure 3 (left panel), all the decision guidance approaches reported comparable net benefits to *treat everyone* for threshold probabilities below 15%, and comparable net benefits to *treat no one* for threshold probabilities above 55%. Within the clinical range (from ~15% to ~55%) where *treat no one* and *treat everyone* were not the optimal options, the optimal approaches to decide on OCA prescription were by using total bilirubin values and MELD, as reflected by the higher net benefit curves compared to those observed for albumin, Child‐Pugh score and history of ascites. At a probability threshold of 15 ~ 55%, the approaches including total bilirubin or MELD were associated with significant net reductions of OCA therapies, allowing to save a mean of 29 (*SD* 6) and 31 (*SD* 7) every 100 treatments, without losing any event of OCA response.

## 4 DISCUSSION

Several post‐marketing reports of liver decompensation in patients with PBC cirrhosis on treatment with OCA have been published over the last years.^7^, ^8^, ^9^, ^10^, ^18^, ^19^ Recently, the FDA has restricted the use of OCA in PBC patients with “advanced cirrhosis”, generically defined as those with current or prior evidence of hepatic decompensation or portal hypertension,^10^ and AASLD has recently updated the clinical practice guidelines to incorporate this restriction.^11^ Concerns about safety of OCA calls for a careful assessment of individual risk/benefit before starting treatment with the drug.

In our study, we observed that OCA is still biochemically effective in cirrhotic patients, and approximately one third of them responded according to Poise criteria. However, nearly one out of 10 PBC cirrhotics experienced hepatic SAEs leading to drug withdrawal within the 12 months of OCA treatment. Among the different parameters, we found that elevated serum bilirubin levels at baseline are associated with higher risk of non‐response and hepatic SAEs under exposure to the drug. We have also identified a serum bilirubin level of less than 1.4 mg/dl at baseline as an easy and reliable parameter to discriminate, among cirrhotic patients, those who could safely benefit from OCA therapy. Selecting cirrhotic candidates for OCA therapy is effective in improving the net benefit of the drug by reducing the rate of potentially ineffective and harmful treatments.

PBC cirrhotic patients who do not adequately respond to UDCA represents a difficult‐to‐treat population and with the highest potential benefit from effective therapy. Fibrates do not represent a safe option in this context because of their potential hepatotoxicity.^20^ Therefore, OCA represents the only disease‐modifying therapeutic approach. However, cirrhotic patients were poorly represented in the registrative trial of OCA (POISE), where their number is not entirely clear; indeed, only a subgroup of the subjects enrolled in the study (106, ~49%) underwent screening by transient elastography, among which a minority (20, ~19%) showed a value indicative of cirrhosis (≥16.9 kPa).^5^ They reported only one case of ascitic decompensation and one of hepatic encephalopathy (both in the 5–10 mg titrating arm), among the serious adverse events observed within the first 12 months. In the 3‐year interim analysis of the open‐label extension of the study, further hepatic SAEs were observed, including other eight episodes of ascites and three cases of variceal haemorrhage, mostly in patients with documented baseline liver cirrhosis.^6^ Recently published real‐world studies, including our own, have assessed the efficacy and safety of OCA,^7^, ^8^, ^9^ and the efficacy of OCA and fibrates,^8^, ^9^ in PBC national cohorts. In our study, we already reported some cases of hepatic SAEs in cirrhotic patients,^7^ which were observed also in the Iberian and Canadian cohorts.^8^, ^9^ However, the limited number of cirrhotic patients in these cohorts hampered the possibility to analyse in depth their specific safety profile with the drug. Eaton J et al described a small case series of 6 PBC patients started on OCA who experienced worsening of liver function, and all but two had total bilirubin >2 x ULN before treatment.^18^ John B et al recently investigated the effect of OCA in a retrospective cohort using national data from US veterans including PBC cirrhotic patients of whom 21 were on OCA and 84 were not on OCA.^19^ Using a propensity score model, OCA use was associated with an increased risk of hepatic decompensation (adjusted hazard ratio, 3.9; 95% confidence interval, 1.33–11.57), while they found no association between OCA use and liver‐related mortality or transplantation.^19^ However, these findings are limited by the reduced sample size, and by the fact that the analysed cohort of Veterans includes more males than the traditional PBC cohorts. As such, the results obtained are at most generalizable to a population of white, male individuals.

This study from the Italian PBC registry focused on PBC patients with cirrhosis under OCA therapy. We confirmed that the effect of the drug, in terms of reduction of ALP and ALT levels and stabilization of bilirubin value, is maintained also in the cirrhotic stage of disease. By applying the widely used *Poise criteria* of response, 32% of patients in the overall cohort achieved a response at 12 months; however, when considering patients completing the treatment period, the rate was 41%. Considering that in Italy OCA is actually indicated in patients with ALP/ULN≥1.5, these rates were shown to be consistent also in the subset of individuals with ALP/ULN≥1.67, that is, after excluding those with ALP/ULN 1.5–1.67 that, as such, could have favoured higher response rates. By applying the *normal range criteria*, which have been recently proposed as the gold standard for treatment endpoint in PBC, only 9% of patients achieved a response in the overall population and 11.5% when considering only patients completing the treatment period. To note, consistent with our previous data, the desirable goal of normalizing liver biochemistry is reached by a minority of PBC patients within the first 12 months of OCA treatment, independently from the presence of liver cirrhosis.^7^

In this study, out of 100 patients prospectively analysed, 22 patients dropped out treatment and the majority (68%) between 6 and 12 months. Ten suspended the drug because of itch, while 9 (41%) because of hepatic SAEs, with 5 patients experiencing worsening of liver function and/or ascitic decompensation, 3 upper digestive bleeding and 1 patient dying after TIPS placement for upper digestive bleeding. Moreover, we highlighted a panel of clinical/biochemical parameters that were found to be significantly associated with non‐response and to efficiently predict the on‐treatment occurrence of hepatic SAEs, such as baseline values of total bilirubin, albumin, INR, Child‐Pugh and MELD scores and history of ascites. We suggest these variables should be carefully evaluated by treating physicians when selecting cirrhotic candidates to OCA therapy in order to maximize the chance of achieving biochemical response and limiting the occurrence of hepatic SAEs. Interestingly, in contrast with the recent dictation from FDA incorporated in the latest PBC guidelines,^11^ surrogate markers of portal hypertensions such as platelet count and the presence of oesophageal varices were not associated with the risk of hepatic SAEs under OCA. Indeed, relying only on these parameters would have led to restrict OCA therapy to a sizeable number of patients potentially benefitting from the drug (those with bilirubin <1.4 mg/dl), and, even most importantly, to allow some high‐risk patients (those with bilirubin ≥1.4 mg/dl) to begin OCA treatment. This is consistent with preclinical studies in rat models of cirrhotic portal hypertension, which suggested an even beneficial effect of OCA on portal hemodynamics.^21^

Among all, total bilirubin ≥1.4 mg/dl was the most accurate biochemical predictor of hepatic SAEs, showing high specificity (88%), NPV (96%) and general accuracy (86%), despite limited PPV (35%). Therefore, while treating PBC cirrhotic individuals above this cut‐off is not always associated with hepatic SAEs, treating those below it will hardly translate into the occurrence of these poor outcomes. Notably, a higher bilirubin level was also predictor of non‐response to OCA treatment. Consistently, the importance of total bilirubin in the decision‐making on OCA treatment was confirmed by means of DCA, which analysed the impact of the different predictors on clinical consequences. The model including total bilirubin conferred the highest net benefit across a wide range of clinical scenarios, both compared to other parameters and to the current default strategy of patients' selection for OCA. Indeed, DCA results suggest that selecting cirrhotic PBC candidates upon baseline total bilirubin level could potentially lead to an improved OCA effectiveness by avoiding around 30 potentially ineffective and dangerous treatment every 100.

The potential mechanism/s behind OCA toxicity are not clear. Whether this relates to the role of Farnesoid X receptor (FXR) agonist in the rate‐limiting steps in bile acid synthesis and in the feedback loop of bile acid homeostasis or to its pleiotropic role in the regulation of numerous metabolic pathways can be only speculative and it is worth to be explored. Serum bilirubin, marker of liver synthetic function, was predictive of hepatic SAEs. OCA has been recently shown to increase hepatic blood perfusion and the hepatic transport of the conjugated bile acids from hepatocytes into biliary canaliculi.^22^ This mechanism in patients with advanced disease might be clogged up by hepatocyte failure and ductopenia, as expressed by elevated serum bilirubin. Consistently, OCA‐induced choleresis has been proven toxic in conditions of impaired biliary outlet, such as in extrahepatic bile duct obstruction.^23^

This study has some limitations. Without a control cohort, this study can neither assess if the risk of hepatic SAEs in this cohort of PBC cirrhotic patients was increased by OCA administration, nor verify the potential determinants of OCA toxicity. However, we were mainly interested in verifying predictors of non‐response to OCA and of hepatic SAEs under treatment (regardless whether induced by OCA or not), therefore providing clinicians with easy indicators to rely on when selecting OCA cirrhotic candidate trying to avoid an ineffective and potentially harmful therapy. Moreover, by this time, the presence of an approved second‐line treatment renders the availability of a control group unethical/unfeasible. Therefore, we focused on the SAEs leading to OCA discontinuation, not considering all AEs occurred: this is a limitation if we consider the AE leading to dose reduction are not reported in the present study. The cohort size is only relatively sizeable; however, based on previous experiences already published it is unlikely to have larger cohorts of cirrhotic PBC patients to analyse, particularly after the recent FDA warning on the use of OCA in patients with advanced disease.

In conclusion, our data confirm that OCA is effective in almost 30% of cirrhotic patients; elevated baseline bilirubin is predictive of hepatic SAEs and of treatment failure to OCA. A baseline assessment of total bilirubin ≥1.4 mg/dl should discourage the use of OCA.

## CONFLICT OF INTEREST

UVG received speaking fees from Intercept, Shionogi, MSD and Gilead. MC consults for and advices for Intercept, Cymabay, Moderna, Perspectum, Albireo, Echosense, Mayoly. All other authors deny any personal and financial conflict of interests with the present work.

## Supporting information


**Data S1** Supporting information

## APPENDIX A

Collaborators

Valentina Feletti, Alessandro Mussetto (Gastroenterology Unit, Santa Maria Delle Croci Hospital, Ravenna, Italy), Rosanna Venere (Department of Translational and Precision Medicine, University La Sapienza, Rome, Italy), Giulia Bernaccioni (Unit of Clinical Medicine and Hepatology, Campus Bio‐Medico University of Rome), Marie Graciella Pigozzi (Gastroenterology Unit, Spedali Civili Hospital, Brescia, Italy), Stefano Fagiuoli (Hepatology and Liver Transplant Unit, Papa Giovanni XXIII Hospital, Bergamo, Italy), Natalia Terreni (Hepatology Unit, Valduce Hospital, Como, Italy), Pietro Pozzoni (Hepatology Unit, Alessandro Manzoni Hospital, Lecco, Italy), Leonardo Baiocchi, Giuseppe Grassi (Hepatology Unit, University of Rome “Tor Vergata”, Rome, Italy), Maria Vinci (Hepatology Unit, Niguarda Hospital, Milan, Italy), Valentina Bellia (Hepatology Unit, Valtellina e Alto Lario Hospital, Sondrio, Italy), Roberto Boldizzoni (Hepatology Unit, Treviglio Caravaggio Hospital, Treviglio, Italy), Silvia Casella(Hepatology Unit, Spedali Civili Gardone Val Trompia, Brescia, Italy), Barbara Omazzi (Gastroenterology Unit, Guido Salvini Hospital, Rho, Italy), Guido Poggi (Oncology Unit, Istituto di Cura Città di Pavia, Pavia, Italy).
