# Supplementary material for: Predictors of serious adverse events and non‐response in cirrhotic patients with primary biliary cholangitis treated with obeticholic acid
Source: Liver Int. 2022 Aug 23;42(11):2453–65. doi: 10.1111/liv.15386 (PMC9804305; doi:10.1111/liv.15386)

**Supplementary Materials**

**Predictors of Serious Adverse Events and Non-response in Cirrhotic Patients With Primary Biliary Cholangitis treated with Obeticholic Acid**

Summary

[Supplementary methods 2](#_Toc100499987)

[Decision Curve Analysis (DCA) 2](#_Toc100499988)

[Supplementary Results 4](#_Toc100499989)

[Discontinuation of OCA for hepatic SAEs. 4](#_Toc100499990)

[Supplementary table 1. Baseline characteristic of subjects discontinuing OCA for hepatic SAEs. 5](#_Toc100499991)

[Supplementary Figure 1. Rates of response to OCA therapy according to the POISE (left panel) and the normal range criteria (right panel) in the 79 patients with ALP/UNL≥1.67, of the Overall Cohort and Treatment Completers Cohort. p>0.05 for all comparisons with OCA response rate in the complete cohort of 100 PBC cirrhotic patients. 7](#_Toc100499992)

[Supplementary Figure 2. Variation of alkaline phosphatase, alanine aminotransferase and total bilirubin during Obeticholic acid treatment. Data is reported as median values with interquartile range at different time point. The p values are from the Wilcoxon test for paired observations. *, p<0.05; **, p<0.01; ULN, upper limit of normal. 8](#_Toc100499993)

[Supplementary figure 3. Rates of response to OCA therapy according to the POISE (left panel) and the normal range criteria (right panel) in the 90 patients under OCA and UDCA therapy (not taking also Bezafibrate), of the Overall Cohort and Treatment Completers Cohort. p>0.05 for all comparisons with OCA response rate in the complete cohort of 100 PBC cirrhotic patients. 9](#_Toc100499994)

# Supplementary methods

## Decision Curve Analysis (DCA)

Diagnostic and prognostic models are typically evaluated with measures of accuracy that do not address clinical consequences, such as the benefits or harms of a medical intervention, including also costs, availability, and patients’ willingness to be treated. Decision-analytic techniques allow assessment of such outcomes but often require collection of additional information and may be cumbersome to apply to models that yield a continuous result. Decision curve analysis (DCA) was introduced in 2006 by Vickers (Vickers AJ, Med Decis Making. 2006;26(6):565–74), and is a method for evaluating and comparing prediction models that incorporates clinical consequences, requires only the data set on which the models are tested, and can be applied to models that have either continuous or dichotomous results. The method sought to overcome the limitations of both traditional statistical metrics, such as discrimination and calibration, which are not directly informative as to clinical value, and full decision analytic approaches, which are too unwieldy to be used in regular biostatistical practice.

In brief, DCA calculates a clinical NET BENEFIT for one or more prediction models or diagnostic tests (*grey and dashed lines*) as compared to two default strategies: 1) TREAT ALL (*solid line*): assume that all patients are test positive and therefore treat everyone, or 2) TREAT NONE (*thick solid line*): assume that all patients are test negative and offer treatment to no one.

The NET BENEFIT is computed and plotted across a range of THRESHOLD PROBABILITIES, defined as the minimum probability of treatment success that can be accepted by the treating physician and by the patient.

By definition, the threshold probability at the intersection of the TREAT ALL and the TREAT NONE lines represent the baseline probability of treatment success without implementing any other parameter in the decision-making strategy.

Accordingly, the clinical scenario drives the choice of the most convenient threshold probability. In particular:

- low threshold probabilities are assumed in situations when a low treatment success could be accepted, i.e. when the patient is willing to be treated (compliant patients), or the treated disease is severe, or the treatment has a low rate of serious adverse events, or the treatment is cheap…

- high threshold probabilities are conversely assumed in situations when a low treatment success cannot be accepted, i.e. when the patient is worried about treatment (non-compliant patients), or the treated disease is mild, or the treatment has moderate to high rate of serious adverse events, the treatment is expensive…

The BEST STRATEGY is that showing the highest NET BENEFIT (highest curve) across the widest possible range of THRESHOLD PROBABILITIES. Note that a single strategy may be superior only to a specific subset of probabilities. The clinical meaning of the THRESHOLD PROBABILITIES, and which ranges are to be considered, should be decided considering the specific clinical setting, *i.e.* no fixed values are suggested.

# Supplementary Results

## Discontinuation of OCA for hepatic SAEs.

Within the study period 9 patients discontinued OCA due to hepatic SAEs. Here, we report a short description of each subject:

- ID 9 was a Child-Pugh A5 woman who initiated OCA and subsequently discontinued after 9 months for severe anemization due to bleeding from portal hypertensive gastropathy. Data concerning upper endoscopy before OCA start were not available.

- ID 16 was a Child-Pugh A5 woman who initiated OCA and suspended it after 5 months for worsening bilirubin levels (from 1.09 mg/dL to 14 mg/dL).

- ID 20 was a Child-Pugh A5 cirrhotic woman who initiated OCA on July 2019 at the dose of 5 mg/day. On May 2019, an upper endoscopy had been negative for gastro-oesophageal varices. Al the 9^th^ month of OCA treatment, she experienced massive bleeding from F2 oesophageal varices, and underwent multiple endoscopic band ligations. OCA treatment was suspended.

- ID 35 was a Child-Pugh A6 man who initiated OCA and experienced severe pruritus, malaise and worsening bilirubin levels within one month from OCA start (from 1.6 mg/dL to 4.5 mg/dL), leading to treatment discontinuation.

- ID 65 was a Child-Pugh B9 cirrhotic woman, who initiated OCA at the dose of 5 mg/week, discontinued OCA after four months for worsening bilirubin (from 1.8 mg/dL to 8 mg/dL). After a partial recovery of liver function, she progressively deteriorated and received a liver transplant seven months after OCA withdrawal.

- ID 69 was a Child-Pugh A5 cirrhotic woman who died within the study period for liver failure after a TIPS placement for refractory bleeding from portal hypertensive gastropathy. Data concerning upper endoscopy before OCA start were not available.

- ID 82 was a Child-Pugh A5 woman who discontinued OCA after 3 months of treatment for bleeding from F3 gastro-oesophageal varices. An upper endoscopy performed about 6 months before OCA start had reported F1 oesophageal varices.

- ID 107 was a Child-Pugh B8 woman who initiated OCA and subsequently developed diuretic-intractable ascites and spontaneous bacterial peritonitis, leading to OCA discontinuation. She had no previous history of ascites.

- ID 108 was a Child-Pugh B7 woman who initiated OCA and subsequently discontinued for the development of relapsing hydrotorax, hepato-renal syndrome and jaundice. She had history of previous ascites, which was however in remission with diuretics at the time of OCA start.

## Supplementary table 1. Baseline characteristic of subjects discontinuing OCA for hepatic SAEs.

| **Characteristic** | **Hepatic Severe Adverse Events** | |  |
| --- | --- | --- | --- |
|  | **NO**  N = 91 | **YES**  N = 9 | **p** |
|  |  |  |  |
| ***Socio-demographics and comorbidities*** |  |  |  |
| Sex, female | 87 (96%) | 8 (89%) | 0.4 |
| Age at OCA start, years | 62 (54, 67) | 60 (56, 72) | >0.9 |
| Age at PBC diagnosis, years | 51 (43, 57) | 54 (43, 55) | 0.8 |
| Duration of disease before OCA start, years | 9 (4, 16) | 11 (6, 13) | 0.8 |
| Body Mass Index, Kg/m^2 | 24.7 (22.0, 27.2) | 24.1 (22.2, 24.7) | 0.4 |
| Diabetes | 11 (12%) | 1 (11%) | >0.9 |
|  |  |  |  |
| ***Liver disease characterization*** |  |  |  |
| AMA positivity | 76 (84%) | 7 (78%) | 0.6 |
| ANA positivity | 46 (51%) | 6 (67%) | 0.5 |
| PBC-AIH overlap | 13 (14%) | 1 (11%) | >0.9 |
| Diagnosis of cirrhosis |  |  | >0.9 |
| *Clinical* | 61 (68%) | 7 (78%) |  |
| *Histological* | 22 (24%) | 2 (22%) |  |
| *Elastographic^*^* | 7 (7.8%) | 0 (0%) |  |
| Child-Pugh class |  |  | <0.001 |
| *A* | 91 (100%)*^$^* | 6 (67%)*^$$^* |  |
| *B* | 0 (0%) | 3 (33%)*^$$$^* |  |
| *C* | 0 (0%) | 0 (0%) |  |
| MELD | 6.78 (6.43, 8.20) | 8.51 (7.70, 12.97) | 0.009 |
| Ascites |  |  | 0.005 |
| *Absent* | 89 (98%) | 6 (67%) |  |
| *Controlled with diuretics* | 2 (2.2%) | 2 (22%) |  |
| *Present* | 0 (0%) | 1 (11%) |  |
| Encephalopathy | 0 (0%) | 0 (0%) |  |
| Esophageal varices, presence | 26 (29%) | 5 (56%) | 0.13 |
|  |  |  |  |
| ***OCA therapy*** |  |  |  |
| Indication to OCA start |  |  | >0.9 |
| *UDCA intolerance* | 0 (0%) | 0 (0%) |  |
| *Inadequate response to UDCA* | 91 (100%) | 9 (100%) |  |
| *acc. to Paris I criteria* | 53 (58%) | 7 (78%) | 0.3 |
| *acc. to Paris II criteria* | 89 (98%) | 9 (100%) | >0.9 |
| *acc. to Toronto criteria* | 72 (79%) | 7 (78%) | >0.9 |
| OCA regimen |  |  | 0.5 |
| *5 mg 3 times a week uptitrated to 5 mg daily* | 3 (3.3%) | 0 (0%) |  |
| *5 mg daily* | 58 (64%) | 7 (78%) |  |
| *5 mg daily uptitrated to 10 mg* | 19 (21%) | 1 (11%) |  |
| *5 mg every other day* | 1 (1.1%) | 1 (11%) |  |
| *5 mg every other day uptitrated to 5 mg daily* | 4 (4.4%) | 0 (0%) |  |
| *5 mg twice a week* | 2 (2.2%) | 0 (0%) |  |
| *5 mg weekly* | 4 (4.4%) | 0 (0%) |  |
|  |  |  |  |
| ***Concomitant/Previous therapies*** |  |  |  |
| UDCA dose, mg/kg | 15.00 (15.00, 17.07) | 15.50 (15.00, 16.98) | 0.7 |
| Fibrate therapy |  |  | 0.7 |
| *NO* | 73 (80%) | 8 (89%) |  |
| *Before and stopped before OCA start* | 8 (8.8%) | 0 (0%) |  |
| *Before and continued during OCA therapy* | 6 (6.6%) | 1 (11%) |  |
| *After OCA start* | 3 (3.3%) | 0 (0%) |  |
| *After OCA discontinuation* | 1 (1.1%) | 0 (0%) |  |
|  |  |  |  |
| ***Biochemical*** |  |  |  |
| ALP/ULN at baseline | 2.05 (1.73, 2.83) | 2.62 (1.70, 3.32) | 0.5 |
| ALT/ULN at baseline | 1.03 (0.75, 1.65) | 1.45 (1.03, 2.50) | 0.11 |
| AST/ULN at baseline | 1.20 (0.90, 1.77) | 1.40 (1.18, 2.63) | 0.10 |
| GGT/ULN at baseline | 4.3 (2.6, 7.2) | 4.8 (4.2, 6.4) | 0.6 |
| Total Bilirubin/ULN at baseline | 0.90 (0.69, 1.10) | 1.40 (1.09, 1.60) | 0.015 |
| Platelets (x10^9^/L) | 152 (120, 200) | 169 (117, 231) | 0.5 |
| Albumin, g/dL | 4.00 (3.61, 4.29) | 3.30 (3.10, 3.62) | 0.003 |
| INR | 1.00 (0.97, 1.07) | 1.12 (1.00, 1.26) | 0.050 |
| Creatinine, mg/dL | 0.70 (0.60, 0.81) | 0.70 (0.68, 0.80) | 0.7 |

Data reported as median with interquartile range or as numbers with percentages. Comparisons were carried out by means of chi-squared test or Wilcoxon test. *Fibroscan > 16.9 KPa. ^$^Out of 91 subjects with Child-Pugh class A, 76 and 15 had a Child-Pugh score of 5 and 6, respectively. *^$$^* Out of 6 subjects with Child-Pugh class A, 4 and 2 had a Child-Pugh score of 5 and 6, respectively. *^$$$^* Out of 3 subjects with Child-Pugh class B, 1, 1 and 1 had a Child-Pugh score of 7, 8 and 9, respectively. Paris I criteria: ALP <3x ULN, ALT <2x ULN and bilirubin <1 mg/dl. Paris II criteria: ALP <1.5x ULN, ALT <1.5x ULN and bilirubin <1 mg/dl. Toronto criteria: ALP <1.67x ULN.

*Abbreviations:* Acc, according; AIH, autoimmune hepatitis; ALP, alkaline phosphatase; ALT, alanine transferase; AMA, antimitochondrial antibodies; ANA, antinuclear antibodies; AST, aspartate transferase; GGT, gamma-glutamyl transferase; INR, international normalized ratio; MELD, model for end stage liver disease; PBC, primary biliary cholangitis; UDCA, ursodeoxycholic acid; ULN, upper limit of normal.

## Supplementary Figure 1. Rates of response to OCA therapy according to the POISE (left panel) and the normal range criteria (right panel) in the 79 patients with ALP/UNL≥1.67, of the Overall Cohort and Treatment Completers Cohort. p>0.05 for all comparisons with OCA response rate in the complete cohort of 100 PBC cirrhotic patients.

**
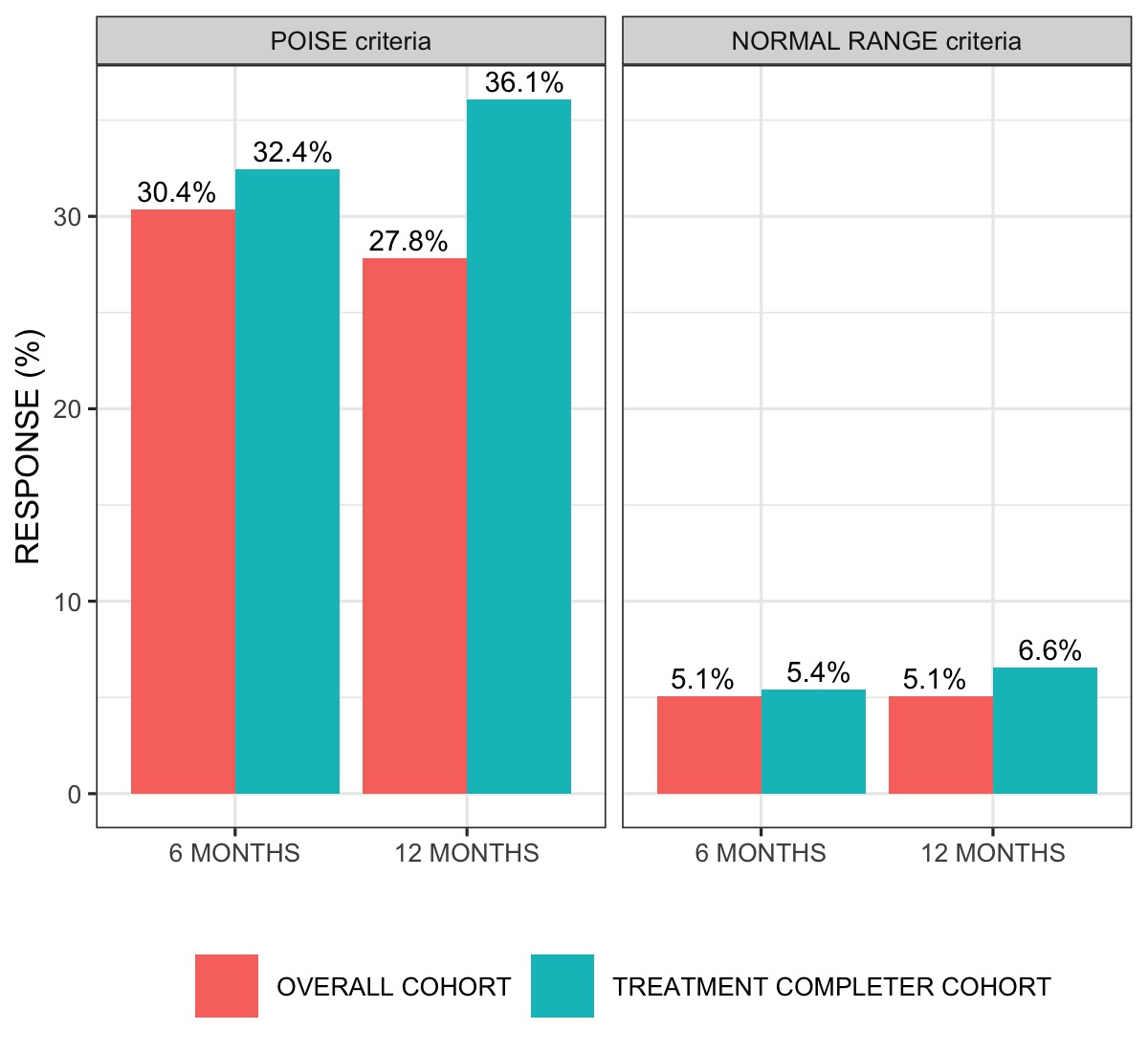
**

## Supplementary Figure 2. Variation of alkaline phosphatase, alanine aminotransferase and total bilirubin during Obeticholic acid treatment. Data is reported as median values with interquartile range at different time point. The p values are from the Wilcoxon test for paired observations. *, p<0.05; **, p<0.01; ULN, upper limit of normal.


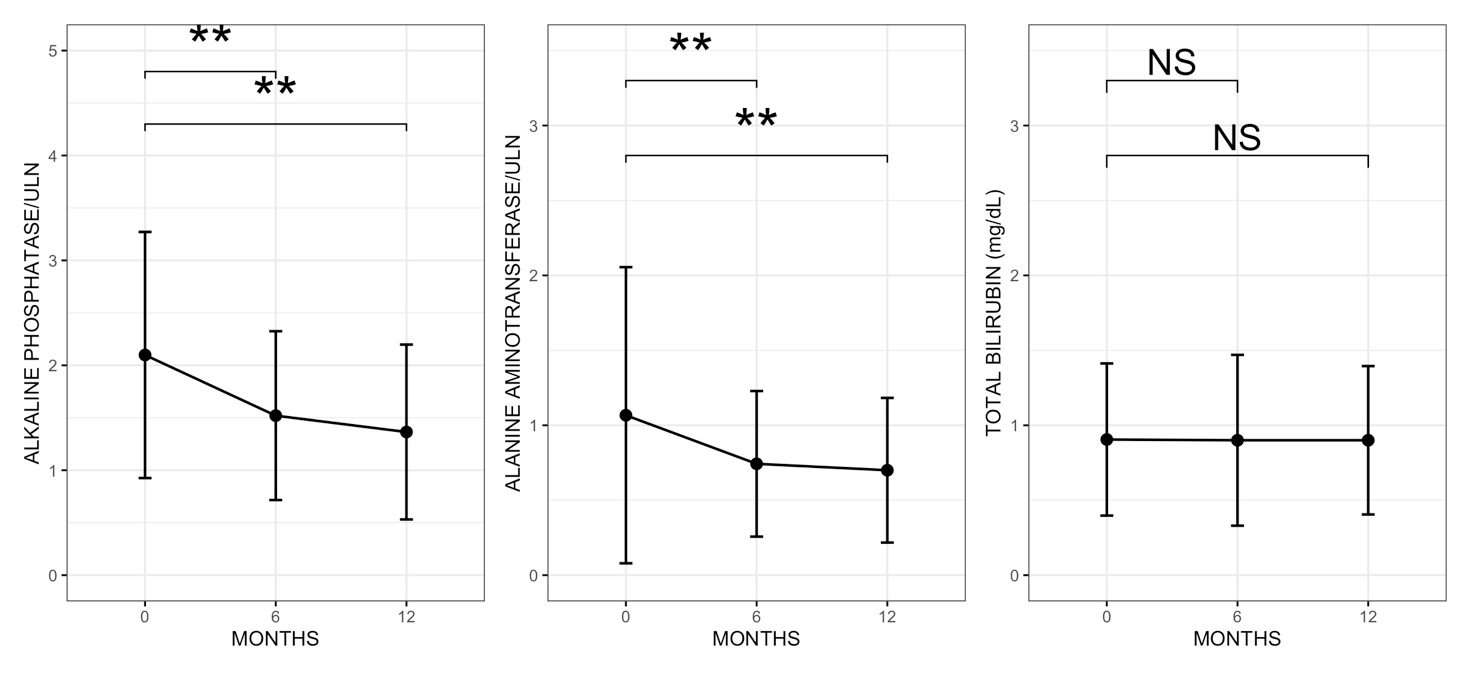


## Supplementary figure 3. Rates of response to OCA therapy according to the POISE (left panel) and the normal range criteria (right panel) in the 90 patients under OCA and UDCA therapy (not taking also Bezafibrate), of the Overall Cohort and Treatment Completers Cohort. p>0.05 for all comparisons with OCA response rate in the complete cohort of 100 PBC cirrhotic patients.


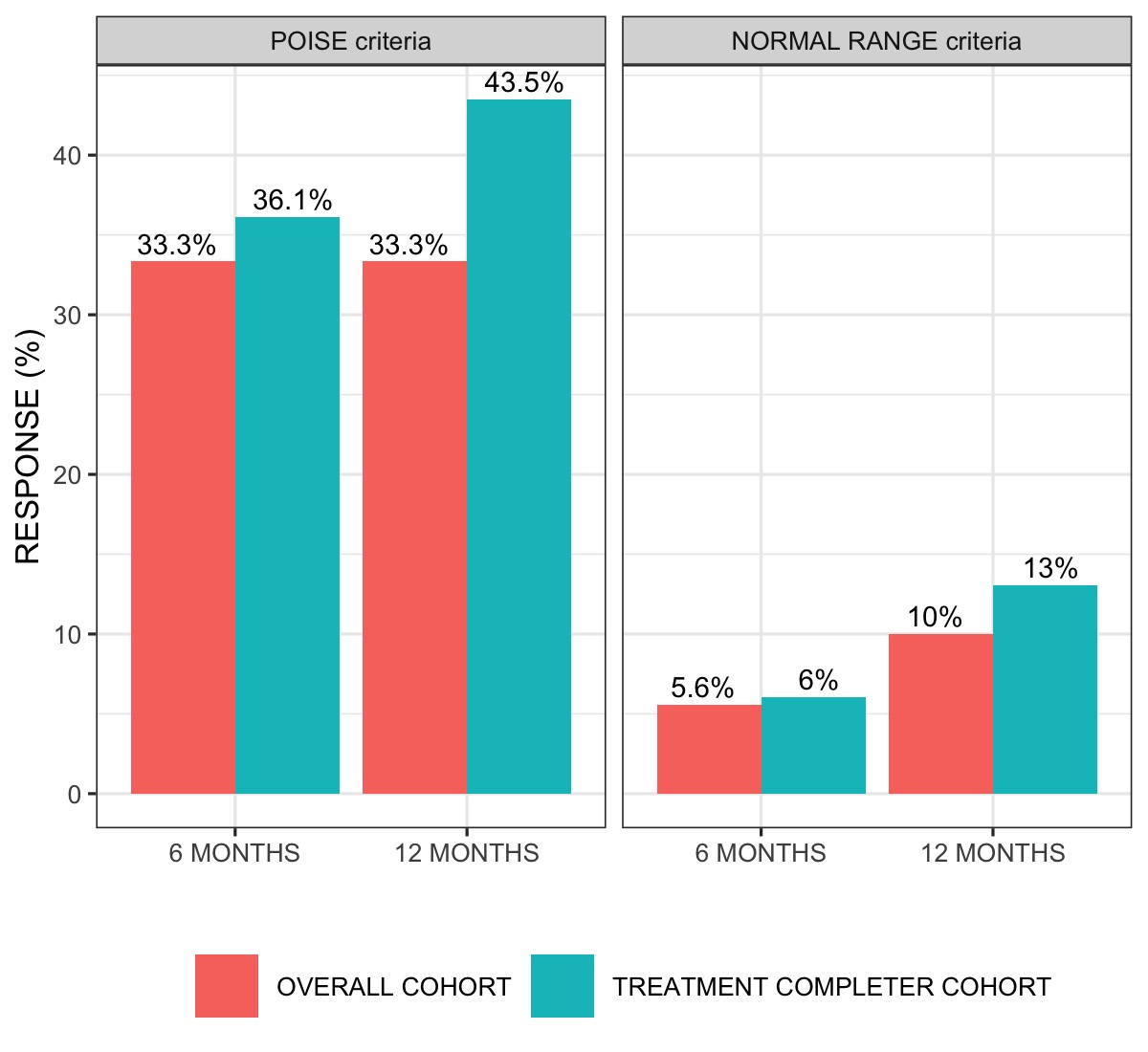

Supplement: Supplementary file 1 — Data S1 Supporting information [file LIV-42-2453-s001.docx]
